# Effective Communication of Personalized Risks and Patient Preferences During Surgical Informed Consent Using Data Visualization: Qualitative Semistructured Interview Study With Patients After Surgery

**DOI:** 10.2196/29118

**Published:** 2022-04-29

**Authors:** Undina Gisladottir, Drashko Nakikj, Rashi Jhunjhunwala, Jasmine Panton, Gabriel Brat, Nils Gehlenborg

**Affiliations:** 1 Department of Biomedical Informatics Harvard Medical School Harvard University Boston, MA United States; 2 Department of Surgery Beth Israel Deaconess Medical Center Boston, MA United States; 3 Geisel School of Medicine Dartmouth College Hanover, NH United States

**Keywords:** data visualization, surgical informed consent, shared decision-making, biomedical informatics

## Abstract

**Background:**

There is no consensus on which risks to communicate to a prospective surgical patient during informed consent or how. Complicating the process, patient preferences may diverge from clinical assumptions and are often not considered for discussion. Such discrepancies can lead to confusion and resentment, raising the potential for legal action. To overcome these issues, we propose a visual consent tool that incorporates patient preferences and communicates personalized risks to patients using data visualization. We used this platform to identify key effective visual elements to communicate personalized surgical risks.

**Objective:**

Our main focus is to understand how to best communicate personalized risks using data visualization. To contextualize patient responses to the main question, we examine how patients perceive risks before surgery (research question 1), how suitably the visual consent tool is able to present personalized surgical risks (research question 2), how well our visualizations convey those personalized surgical risks (research question 3), and how the visual consent tool could improve the informed consent process and how it can be used (research question 4).

**Methods:**

We designed a visual consent tool to meet the objectives of our study. To calculate and list personalized surgical risks, we used the American College of Surgeons risk calculator. We created multiple visualization mock-ups using visual elements previously determined to be well-received for risk communication. Semistructured interviews were conducted with patients after surgery, and each of the mock-ups was presented and evaluated independently and in the context of our visual consent tool design. The interviews were transcribed, and thematic analysis was performed to identify major themes. We also applied a quantitative approach to the analysis to assess the prevalence of different perceptions of the visualizations presented in our tool.

**Results:**

In total, 20 patients were interviewed, with a median age of 59 (range 29-87) years. Thematic analysis revealed *factors that influenced the perception of risk (the surgical procedure, the cognitive capacity of the patient, and the timing of consent;* research question 1); *factors that influenced the perceived value of risk visualizations (preference for rare event communication, preference for risk visualization, and usefulness of comparison with the average;* research question 3); and perceived usefulness and use cases of the visual consent tool (research questions 2 and 4). Most importantly, we found that patients preferred the visual consent tool to current text-based documents and had no unified preferences for risk visualization. Furthermore, our findings suggest that patient concerns were not often represented in existing risk calculators.

**Conclusions:**

We identified key elements that influence effective visual risk communication in the perioperative setting and pointed out the limitations of the existing calculators in addressing patient concerns. Patient preference is highly variable and should influence choices regarding risk presentation and visualization.

## Introduction

### Background

In the United States, >50 million surgical procedures are performed annually [1]. For each procedure, a clinician obtains informed consent from the patient or a surrogate. The discussion during this process plays an important legal and ethical role and should determine the appropriate treatment plan for each patient. The literature suggests that this discussion often does not address the patient’s personal treatment goals [2,3]. In addition, many important details are solely communicated verbally [2]. Unexpected, poorly communicated, or possibly life-threatening events can lead to malpractice lawsuits [2,4,5]. Instead, the informed consent conversation should properly set the patient’s expectations to decrease the chances of what a patient would consider a nonbeneficial outcome [2,3,5].

Although medical professionals agree that determining patient priorities is important for choosing the appropriate treatment plan, the discussion during informed consent often fails to consider the patient’s condition and treatment goals [2,3]. Furthermore, the current informed consent process is not standardized and leaves patients without a clear understanding of the consequences of surgery [6]. There is also a lack of consensus in the medical community regarding which risks to communicate, and risk estimates are often too broad and vary among physicians [2]. Multiple studies have shown that, despite reviewing the surgical procedure and associated risks, the patients’ understanding after these discussions is well below acceptable limits [7]. Risk score calculators try to expand the conversation through personalized risks for any given patient. They provide discrete risk scores for a variety of outcomes based on the surgical procedure and preoperative patient data. Despite the growing prevalence of these tools, the surgical community has not reached a consensus on how to communicate these scores. Some groups have attempted to address this issue by categorizing complications into best case and worst case or good, intermediate, and bad [8-10]. In these approaches, patient preference, which is essential for defining a good outcome for a patient, is not necessarily incorporated or used to inform the conversation.

**Figure 1:**
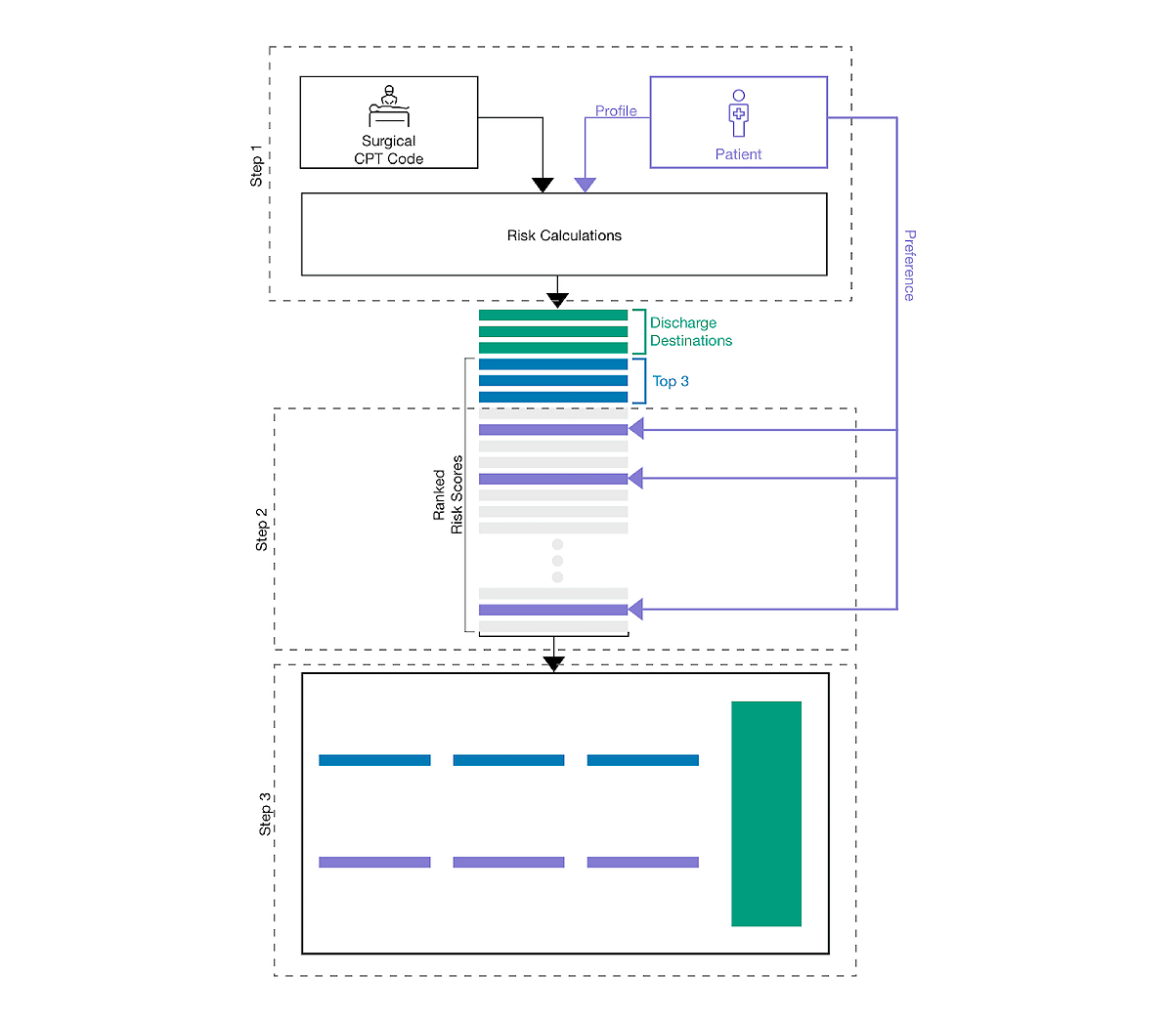
The proposed visual consent tool includes 3 main steps to help the patient and surgeon evaluate the risks of a surgery. In the first step, the personalized risks are calculated using an existing risk model with identification of surgery (eg, Current Procedural Terminology [CPT] code) and patient preoperative data as inputs. The patient then chooses up to 3 risks that are of high concern (purple bars) in addition to the top 3 calculated risks (blue bars). Finally, a visualization of these 6 risks is displayed along with the likelihood of each of the final discharge destinations (green bar).

We propose a design for a visual consent tool to address previous limitations in (1) incorporating patient preferences, (2) setting expectations for the upcoming surgery, and (3) standardizing risk communication during informed consent. The visual consent tool communicates personalized risks to the patients in 3 main steps (Figure 1). First, personalized risks are calculated using one of the risk prediction models currently available [11-14]. These prediction models typically incorporate a surgical Current Procedural Terminology code and patient preoperative data to calculate risks. The design allows for the use of a preferred risk calculator such as the American College of Surgeons (ACS) [13], the Surgical Risk Preoperative Assessment System [12], or the Predictive Optimal Trees in Emergency Surgery Risk [11], among others, without affecting the rest of the workflow. In our particular design instance, we rely on a simulation of the ACS risk calculator at a level that allows us to go through the visual consent tool workflow and conduct our study. Second, patients select a limited number of major concerns (we chose 3 arbitrarily) out of a list of 20 complications produced by the simulated ACS calculator, preranked in descending order of likelihood. Finally, we visualize the probability of the 3 most likely and patient-selected complications as well as the potential discharge destinations: home, rehabilitation, and death. With this, the visual consent tool allows patients to compare the risks of the most likely and prioritized complications and communicates potential discharge destinations after surgery.

### Objectives

Using high-fidelity design mock-ups for the visual consent tool, we conduct a qualitative design feedback study in which we want to address the following research questions: (1) How do patients perceive risks before surgery—does what matters depend on the context? (research question 1), (2) How suitably the visual consent tool is able to present personalized surgical risks—are the patients looking at the risks they really care about the most? (research question 2), (3) How to best communicate these personalized risks using data visualization approaches—are there ways to present these risks that are most understandable to patients? (research question 3), and (4) In which scenarios can the visual consent tool be used, and can it improve the informed consent process—are the patient and surgeon able to engage in a more productive discussion?

Focusing only on the visual consent tool’s personalized risk visualization component (Figure 1, step 3), we conduct semistructured interviews with patients during their postoperative visit to the acute care surgical clinic at an academic medical center. Through thematic analysis of the interviews, we identify several factors that affect the perception of risks and their importance, the perceived value of risk visualization, the preferences for risk visualization, the effects of risk visualization, and the potential usefulness of the visual consent tool in a real-life setting. The report of this study is based on the COREQ (consolidated criteria for reporting qualitative research) guidelines [15].

## Methods

### Visual Consent Tool Design

A schematic overview of the visual consent tool is shown in Figure 1. The visual consent tool consists of three elements: risk calculation, preference identification, and risk visualization.

#### Risk Calculation

Multiple methods for calculating personalized perioperative risks for patients have been published [11-14]. These calculators use collected patient data (eg, age, sex, and smoking status) to calculate the risk of a given postoperative complication.

**Figure 2:**
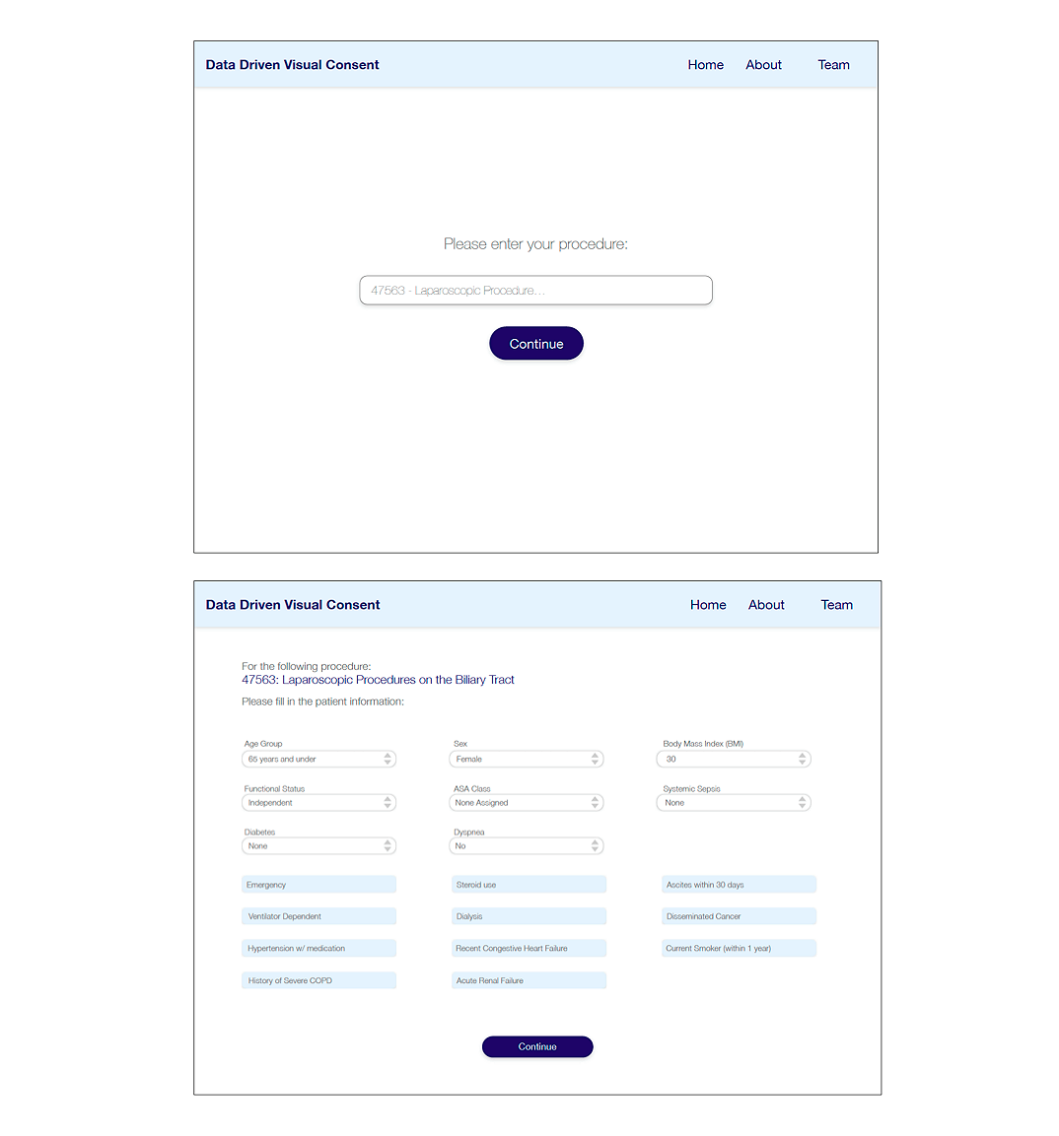
Patient profile and Current Procedural Terminology (CPT) code input. The form at the top is used to enter the CPT code for the surgery, and the form at the bottom is used to provide patient characteristics required by the risk calculator. ASA: American Society of Anesthesia; COPD: chronic obstructive pulmonary disease.

As an example, the ACS risk calculator, the most commonly used tool, leverages National Surgical Quality Improvement Program participant data from >400 hospitals to calculate 20 different perioperative risks [13]. In this study, we did not focus on improving risk calculation. Generally, our approach could be applied to risks calculated using any risk calculator. For practical purposes, we used the results obtained from the ACS risk calculator for this study. In our proposed interface, the surgeon provides information about the surgery by entering a Current Procedural Terminology code and the patient profile (Figure 2).

#### Incorporating Patient Preferences

**Figure 3:**
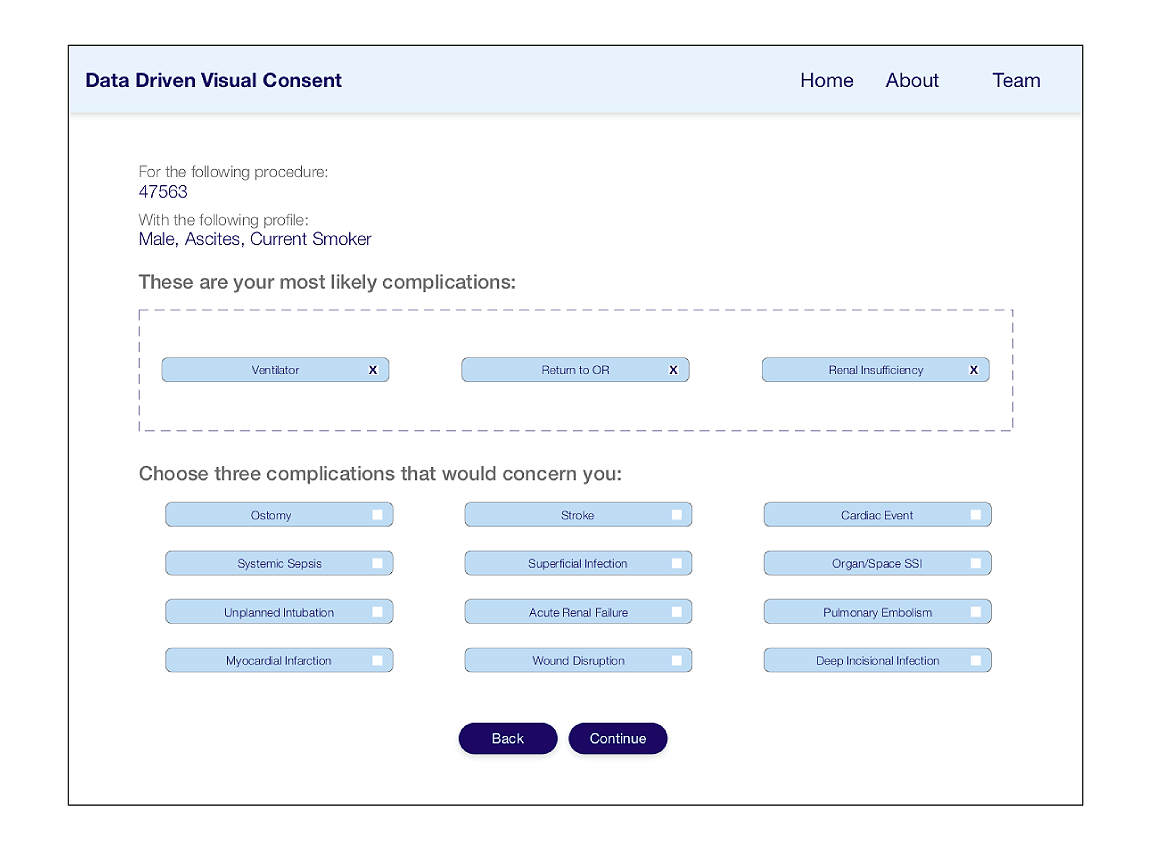
Incorporating patient preferences. A total of 3 most common complications are preselected, with the remaining complications listed in descending order of likelihood. The patient can select up to 3 risks at a time. OR: operating room; SSI: surgical site infection.

To incorporate personal preferences, our tool provides an interface for patients to identify 3 complications that are of particular concern in addition to the top 3 risks that the tool automatically selects as most important based on the risk calculations (Figure 3). After presenting the patient with a list of possible complications preranked by likelihood, patients are able to choose the risks that are most concerning to them (Figure 3).

#### Risk Visualization

**Figure 4:**
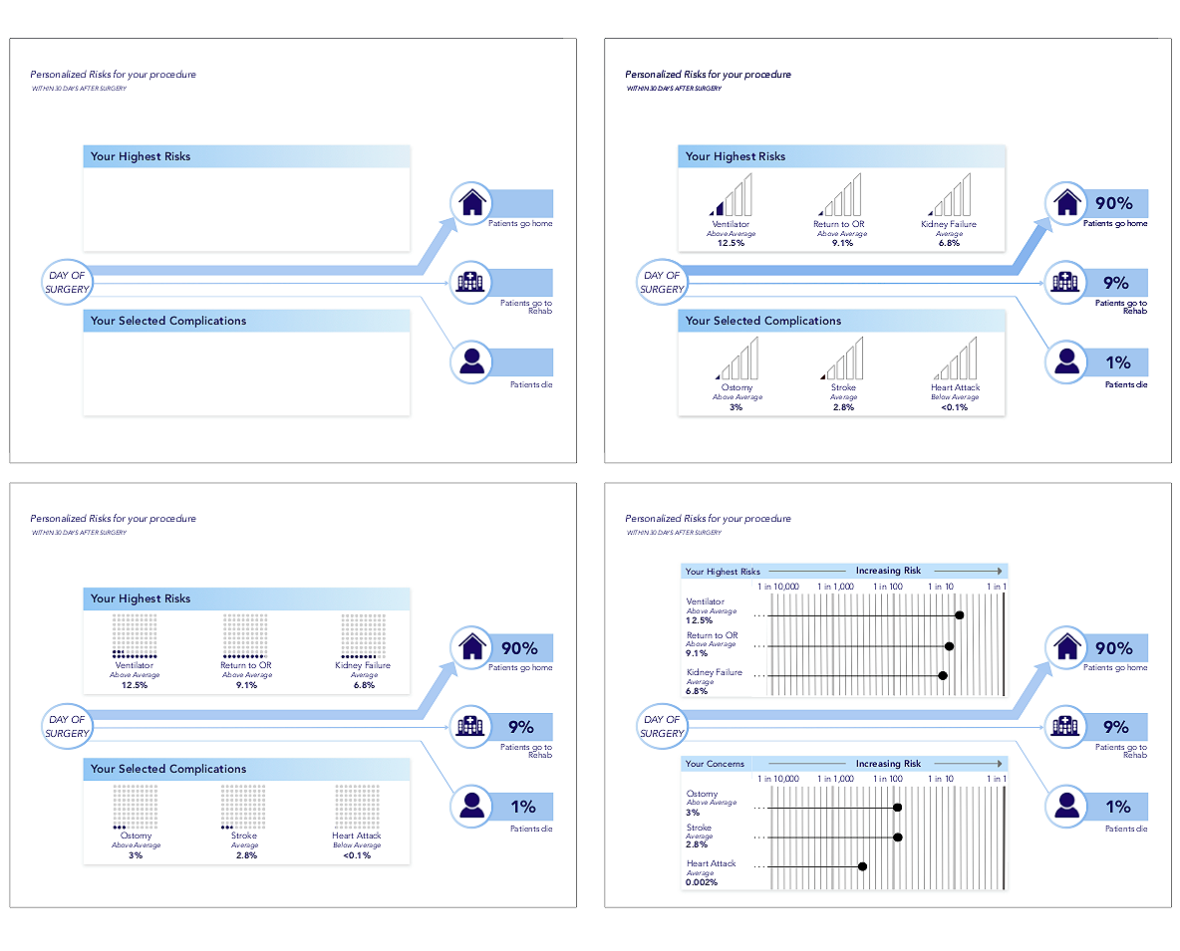
The design of the 3 risk visualizations used in this study. This study investigated 3 visualizations that, through a literature review, were identified as likely to be successful in communicating risks to patients. The top left shows the general layout that all 3 visualizations follow, where the patient’s highest risks are displayed at the top and the complications they have chosen are displayed below to allow for comparison. The likelihood of each discharge destination is separated from the risks and communicated using positional cues and weighted lines. The 3 visualizations tested were bar strength (top right), dot array (bottom left), and logarithmic scale visualization (bottom right). OR: operating room.

The visualization is intended to communicate personalized perioperative risks and the likelihood of the discharge destinations in a clear and understandable manner. The overall goal is to promote a more coherent discussion between the surgeon and patient for improved shared decision-making. The layout includes the most likely preselected complications based on the risk calculations as well as those selected by the patient and the likelihood of each discharge destination (Figure 4, top left). Discharge destinations are communicated using weighted lines to represent likelihood. Preselected and patient-selected complications are boxed separately to allow for comparison between the 2 categories. Given the relatively low rates of complications, the representation of the likelihood of each complication presented a unique challenge that we examined in detail.

**Figure 5:**
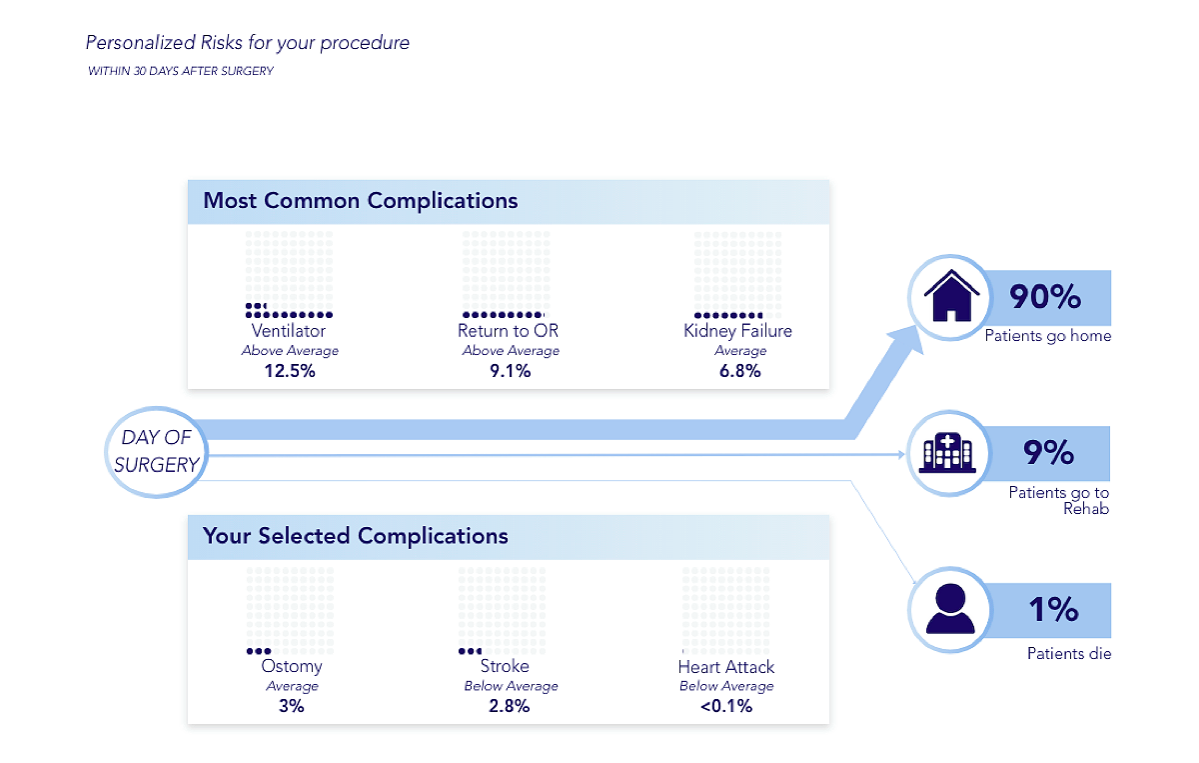
An enlarged image of the final stage of the visual consent tool mock-up used for evaluation with one of the possible visualizations—the dot array. OR: operating room.

We grouped complications into *rare events* (<1%) and *common events* (≥1%). We referred to the Visualizing Health repository [16] to choose visualizations that could be suitable for communicating these events. We chose the *bar strength* visualization that resembles the signal strength on mobile devices and represents a familiar visualization owing to the prevalence of mobile devices (Figure 4, top right). We also chose a waffle chart, called *dot array*, as it is more granular than the bar strength and is recommended by the Visualizing Health repository to accurately communicate risk (Figure 4, bottom left). To be able to more accurately show risks <1% (compared with the *bar strength* and the *dot array*), we chose a logarithmic scale inspired by the perspective scale proposed by Paling [17], which also allows for direct comparison of risks (Figure 4, bottom right). All of the different visualizations—bar strength, dot array, and logarithmic scale—are shown in the context of the final visualization of the visual consent tool. In Figure 5, we present one instance, the dot array, in a larger image for a better presentation of the design.

### Evaluation of the Visual Consent Tool

#### Participants

A convenience sample of 20 patients was interviewed during their postoperative checkup visit to the acute care surgical clinic at an academic hospital. The patients were approached by the interviewer and asked about their willingness to participate in the study. This study only included patients who had undergone a surgical intervention by an acute care surgeon and who agreed to participate with written consent.

#### Ethics Approval

This study was approved by the Beth Israel Deaconess Medical Center Institutional Review Board (2019P000013).

#### Interviewer

The interviews were conducted by the first of the 2 joint first authors of this paper (UG). The researcher had no previous relationship with the participants and briefly stated the purpose of the study at the beginning of the interviews. The interviewer did not have any previous biases aside from the assumption that visualization would be a useful tool for the consent process.

#### Study Procedure

**Figure 6:**
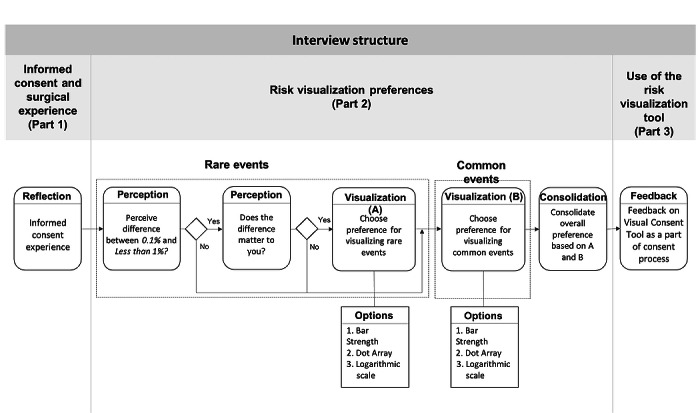
Semistructured interview structure. Part 1 assessed the current informed consent experience. Part 2 assessed risk perception and risk visualization preferences. Part 3 gathered feedback on the useful elements of the visual consent tool and its applicability.

The interview guide was trialed with 2 individuals who were not participants in the study and was refined to fit within 30 minutes and provide answers to our research questions. Figure 6 shows the structure of the semistructured interview (see Multimedia Appendix 1 for the interview guide). The interviews first aimed to understand the patient’s informed consent experience in the current practice—without any visualization aids (Figure 6, part 1 provides answers to research question 1). The second section focused on risk perception and visualization preference (Figure 6, part 2 provides answers to research questions 2 and 3). Finally, the third section assessed perceptions of the value of a visual consent tool and its usefulness during the informed consent process (Figure 6, part 3 provides answers to research question 4). Each of the participants went through the interview only once, and no repeat interviews were conducted.

The interviews were conducted in a clinical setting after a postoperative visit. In some cases, the interviews were conducted in the presence of a significant other or family member of the participant. The interviewer gathered demographic data and impressions of the existing informed consent experience by asking the participants to recall their most recent discussion about the risks of informed consent with a surgeon (Figure 6, part 1).

Following this, the interviewer assessed the patient’s perception of the risk visualizations (Figure 6, part 2). First, using a broadly familiar example, the interviewer evaluated perceptions of life-threatening rare events by asking the patient if there was a notable difference between 0.1% and the phrase *less than 1%* for a likelihood of being struck by lightning. Patients who perceived a difference were shown 3 visualizations and asked to identify the visualization that conveyed most clearly the 0.1% chance of being struck by lightning. Using a similar approach, the interviewer assessed visualization preferences for more common life-threatening events by using an example of a 12.3% chance of an earthquake. Again, the patient was shown the same 3 visualizations and asked to indicate which one conveyed this information best. In cases where the visualizations chosen for rare events differed from the visualizations chosen for common events, the patients chose one of the two preferred options for showing all possible risks: for rare events and more common events.

In the final step (Figure 6, part 3), the final visual consent tool risk and discharge visualization was presented to the patient using the previously selected visualization type. To test the intuitiveness of the design, the patient was asked to explain what they saw and what decisions could be made without receiving any explanation of the final visualization. The interviewer then explained the intended purpose of the visualization and collected additional comments about the visual consent tool.

Finally, the patient was asked to identify situations in which they would find this tool useful, what they found most useful, and what could be improved.

#### Data Analysis

The interviews were recorded using an iPhone (Apple Inc), and no field notes were taken during the interview. Interviews were transcribed using Dragon Dictate 3.0 (Nuance Corporation) with manual verification by the interviewer. Transcriptions were not sent back to the participants for comments and corrections. We conducted a mixed methods analysis of the data. For the qualitative part, the two joint first authors of this paper (UG and DN) conducted a thematic analysis of the data using Microsoft Word and Excel. Each of them inductively coded selections of the transcribed interviews independently by assigning labels to the meaningful discourse units in the patients’ answers. Both researchers reviewed the codes and clarified any disagreements. Codes were collaboratively grouped into categories, and the categories were grouped into themes. These were checked for validity with domain experts and carefully modified to accommodate feedback. We did not conduct any member checks with participants. We also applied a quantitative approach to the analysis and extracted discourse units that expressed different preferences and reasons for those preferences. We used the discourse units identified for each interview to assess the prevalence of different perceptions of the visualizations presented in our visual consent tool.

## Results

### Overview

We interviewed 20 patients attending a postoperative visit. The average age of the cohort was 61.7 (SD 14) years with a range of 29 to 87 (median 59) years, with 55% (11/20) female and 45% (9/20) male participants. The education level ranged from *some high school* to *Ph.D*. Most patients (17/20, 85%) had surgery on the intestines, gallbladder, or appendix, and approximately half of the cases (10/20, 50%) involved emergency procedures.

The thematic analysis of the semistructured interviews revealed three main categories: (1) *factors that influence risk perception* (research question 1), (2) *perceptions of the visualizations* (research questions 2 and 3), and (3) *effects of the proposed visual consent tool and use case scenarios* (research question 4).

### Factors That Influence Risk Perception

#### Overview

We found that patients reacted positively to learning that risks could be personalized. Some stated that personalization of risks was the highlight of the tool as it made them feel more considered as patients. We identified several factors that influenced the perception of those personalized risks, which encompass *the surgical procedure*, *the patient’s cognitive state,* and *the timing of consent*.

#### The Surgical Procedure

Factors that influenced risk perception of the procedure included the clarity of diagnosis, the complexity of the procedure, and the urgency of the case. We found that most patients (17/20, 85%) had a clear diagnosis and were confident in the surgeon’s familiarity with the case. They indicated that they were less concerned about the risks associated with their surgery compared with patients with an unclear diagnosis. The latter group made statements that emphasized the uncertainty of what was about to take place, which increased their anxiety, and made comments such as “[the surgeons] didn’t know what they were getting into.”

Similarly, patients who underwent routine procedures were generally less threatened by the risks compared with patients who were supposed to go through a complex surgical intervention that involved multiple subprocedures. For patients who returned to the operating room, all complications were of low importance, and pain or fear of death outweighed all others.

Finally, patients who had an extended time before the surgery were more willing to analyze the risks and discuss them in greater detail with the surgeon. In contrast, patients who had to go through an emergency surgery were less motivated or even incapable of any form of analysis and were mostly focused on their chance of survival.

#### The Cognitive Capacity of the Patient

We found that the cognitive capacity of the patient, such as the *capacity for unobstructed thinking, medical knowledge,* and *literacy*, played a key role in risk perception*.*

Patients who were in pain or feeling drowsy cared less about complications and wanted to proceed with the surgery as soon as possible.

A consistent theme was the delegation of decision-making to a more medically knowledgeable and literate family member such as a spouse or child when such an opportunity existed. Patients with low health literacy were more likely to not understand the diagnosis and felt that identification of complications was of low importance. These patients would completely delegate the decision-making to the medical professional and restrain themselves from engaging in contributing to the process.

#### The Timing of Consent

The timing of consent varied among patients. For emergency cases, risks were communicated within a few hours of the surgery; for transfer cases or planned operations, risks could have been initially communicated a couple of weeks in advance. For patients with acute conditions, the lists of complications were of low importance as pain and death were described as important factors. Alternatively, patients with subacute conditions (surgeries within 3-24 hours) felt that knowing the risks was important for expectation management.

Interestingly, although knowing about the risks was of varying importance depending on the timing of consent, all respondents were clear that knowledge about the risks would not have influenced their decision to go forward with the surgery.

### Factors That Influence Perceptions of the Visualizations

We identified several factors that influenced the perceptions of the visualizations: *preference for rare event communication, preference for risk visualization,* and *usefulness of comparison with the average.*

#### Preference for Rare Event Communication

Most patients (16/20, 80%) did not have a preference for how rare events were communicated. Patients who wanted to know the exact percentage <1% (3/20, 15%) preferred the logarithmic representation for rare events.

#### Preference for Risk Visualization

Table 1 is based on the preferred visualization after the participant was exposed to the visualization options for rare and common events and asked to consolidate their answers in a single visualization. Table 1 shows the preferences that ranged across the visualizations. Of the three available graphics—the bar strength, dot array, and logarithmic scale—there was no consensus on a preferred visualization.

**Table 1:** Visualization preferences of the patients.^a^

Visualization chosen	Patients, n (%)
Bar strength	5 (25)
Dot array	4 (20)
Logarithmic scale	7 (35)
None	3 (15)
Other	1 (5)

^a^The table shows that the patients had differing preferences for optimal visualization for communicating risks in the visual consent tool. Of the 20 participants, 1 (5%) liked a visual aid but preferred a different visualization from the 3 presented, and 3 (15%) did not express interest in a visual aid.

Patients who preferred the bar strength visualization (5/20, 25%) liked the simplicity and clear step increases, which allowed for quick interpretation. Patients who liked this approach felt that it was less complicated than other options. In addition, 5% (1/20) of patients expressed concern over the discretization of the bars, and a few patients felt that it did not show enough information.

The dot array was endorsed by 20% (4/20) of the patients, who preferred its visual organization and felt that it allowed for comparison of ratios of shaded to grayed out dots. These patients found the dot array easy to understand and that it gave “just the right amount” of information. In addition, 5% (1/20) of patients noted that they would waste time counting dots, and another patient (1/20, 5%) felt that it would be hard to compare risks.

Patients who preferred the logarithmic scale (7/20, 35%) felt that it communicated the risks most clearly and allowed for easy comparison and aggregation of risks. Of those 7 patients, 4 (57%) mentioned that they liked the labeling of *1 in X.* However, patients who did not prefer the logarithmic scale found it the most complicated of the 3 options.

Of the 20 patients, 4 (20%) did not respond positively to the visualizations. In addition, 5% (1/20) of patients was dissatisfied with the choices presented, and 15% (3/20) of participants rejected the visual aids and preferred verbal communication or that the decision be left to the physician. These patients were all aged >75 years, which is notably higher than the average age of the other participants.

#### Usefulness of Comparison With the Average

Most patients (11/20, 55%) expressed indifference to knowing whether their risk was above or below the average. Those who cared about the average stated that it would raise or lower their concern, and some only cared if it was actionable information. Many were not confident in how to include this information in their decision-making process.

### Effects of the Visual Consent Tool and Use Case Scenarios

#### Overall Impression of the Visual Consent Tool

In terms of intuitiveness, most patients (14/20, 70%) found the final visualization intuitive without any context. However, after explaining the context of the tool and the steps leading up to the final visualization, most patients (15/20, 75%) felt able to make decisions with the help of the visualization.

We observed three major effects of the visual consent tool on perceived informed consent: *depth and length of the discussion*, *information retention,* and *risk awareness*.

#### Depth and Length of the Discussion

Most patients (13/20, 65%) stated that the visual consent tool would have helped them pick up more information or be more confident in their surgical decision. Most patients (12/20, 60%) claimed that the visual consent tool would allow them to have a better understanding of the possible complications and their likelihood.

All the patients (20/20, 100%) agreed that the visual consent tool would help stimulate a deeper discussion with their provider. They claimed it would “help [them] think of new questions [they] hadn’t thought of before.”

However, some patients (4/20, 5%) expressed concern that having this information and new questions may extend the discussion and would take too much of the surgeon’s time.

#### Information Retention

A couple of patients (2/20, 10%) also felt that the visualizations might help retain information and suggested using it as a reference to consult after consent.

#### Risk Awareness

Most patients (11/20, 55%) believed that the visual consent tool would make them more aware of potential risks. This made them more confident in their decision to pursue surgery, but most noted that it would not have changed their decision to pursue surgery.

A number of patients (9/20, 45%) noted that it prompted more long-term thinking about what to expect after the surgery and how it would affect not only them but also their families. In addition, the patients expressed concern that this may be too much information for some patients and that it may dissuade them from pursuing a surgery that was in their best interest.

Patients expressed interest in seeing information generally not available in current risk calculators, such as pain level and expected recovery time. Our interviews revealed that the patients were most concerned about their potential health status and whether they would be able to continue normal activity after surgery—including the chances of avoiding an ostomy (Table 2).

**Table 2:** The major concerns of the patients before surgery.^a^

Concern	Patients, n (%)
Ostomy	6 (30)
Health status after the operation	6 (30)
Postoperative plan	5 (25)
Not laparoscopic	4 (20)
Death	4 (20)
Recovery time	3 (15)
General complications	3 (15)
Anesthesia	3 (15)
Pain medication	2 (10)
Life support	1 (5)
Infection	1 (5)
Blood transfusion	1 (5)

^a^This table shows that the patients were most concerned about their potential health status, possible ostomy, and the pain level they could expect after surgery.

## Discussion

### Principal Findings

Essentially, the main purpose of our visual consent tool is to empower patients in the decision-making process, provide them with a degree of control over what is being discussed and how the information is being presented to them, and give them the sense that their voice is being heard. To achieve this, we aimed for a high level of personalization in the design, allowing the patients to not only select the risks they wanted to discuss in depth but also account for different risk visualizations to choose from. This approach is different from the traditional one that positions the surgeon as the sole driver of the discussion regarding the risks and assumes that there is one risk visualization type that is suitable for all patients [12,13,18].

The aforementioned approach allowed us to obtain broad insights into how to tackle the design of visual consent tools. In contrast to existing studies that focus primarily on barriers to tool adoption by surgeons, patients’ perceptions of the material risk communicated by physician-facing risk assessment tools, or the effect of risk visualization on understanding, the study presented here, to our knowledge, is the first to broadly enumerate the requirements and benefits of a personalized visual informed consent tool that incorporates patient-facing risk visualizations and accounts for patient preference for which risks to be visualized and discussed in detail with the surgeon [18-25]. Through our interviews with patients, we elucidated several unique findings that add to the existing literature and inform the present practice of risk communication and the future landscape of personalized risk visualization. First, and perhaps most significantly, the patients did not identify a single preferred risk visualization, and their preferences varied across the 3 visualizations presented. Second, the patients’ concerns regarding postoperative adverse outcomes did not align well with the most probable risks offered by the ACS risk calculator we relied on in our visual consent tool or with the other traditional risk calculators we reviewed. Third, our visual aid was perceived to improve information retention and risk awareness compared with traditional text-only informed consent documents. These findings will be further discussed below in the context of the current literature.

### Variable Preference for Risk Visualization

Almost all patients (15/20, 75%) agreed that the visualizations were useful in communicating risk and would be helpful in their decision-making. Notably, there was no single visualization preferred by most patients, and preferences varied across the available visualizations.

It is difficult to assess whether this finding is aligned with or different from previous findings as most of the literature on this topic of study has evaluated preference for a broad range of visualizations over verbal or textual communication of risks. A few studies have shown that tables, icons, and vertical bar charts are generally preferred over other options such as horizontal bar charts [26,27]. However, the visualizations used in those studies did not cover all 3 visualizations compared in our study.

Although the goal of our study was to determine the preference of patients for risk visualizations, we did not measure understanding quantitatively, though several recent studies have demonstrated differences between participant understanding and preference when presented with different graphical formats to communicate health information [28,29].

Presenting the appropriate risk visualization to a given patient is of high importance but also very challenging. Along these lines, researchers have found that allowing patients to choose a preferred visualization that they feel motivated to interact with versus showing them the useful one that will help them in understanding and using the information better is a decision with trade-offs [30]. To corroborate this, the findings from a study indicated that risks presented in the form of random icons and stacked vertical bar graphs may affect the likelihood of choosing surgery or cause patients to view certain risks as more complex or threatening [31]. Furthermore, a study of 45 adults contemplating the risks and benefits of recombinant tissue plasminogen activator for ischemic stroke concluded that, although patients preferred bar graphs for risk information, accurate recall and confident decision-making decreased when using the bar graph compared with an icon array or stacked graph [29]. In addition, although bar graphs were preferred, patients spent more time studying them compared with the 2 other graphical formats despite these longer decision times correlating with less accurate recall [29]. The question of how to visually present surgical risks to patients is further complicated by our finding that the desire for risk information and involvement in decision-making varies per patient.

In this context, our findings suggest a need for highly tailored patient-facing decision aids with increased flexibility in visualization beyond a one-size-fits-all approach.

### Misalignment Between Patients’ Concerns and Current Risk Calculators

Notably, we found that patient concerns were discordant with the risks presented by traditional risk calculators. According to the interviewed patients, postoperative pain, changes in overall health status, familial burden, and adverse functional outcomes were additional considerations before undergoing a procedure that were very rarely or almost never discussed with them. In comparison, traditional risk calculators highlight major causes of perioperative morbidity, such as the risk of renal failure or venous thromboembolism. If we are to consider surgical risk calculators as a step toward improved shared decision-making, our interviews suggest that it is important for physicians to leverage these tools to communicate the risk of major changes in quality of life, expected functional outcomes, and consequences of the procedure as they are key tenets of informed consent [22,32]. In some cases, patients may consider these risks to outweigh the clinical consequences when considering whether to pursue surgery and, therefore, these risks should be communicated as well and with equal attention. For example, a study showed that 18% of patients with postopen abdominal aortic aneurysm (AAA) repair would not undergo AAA repair again knowing that the recovery process negatively affects functional activity (such as driving and shopping, among other daily tasks), despite understanding the life-threatening consequences of potential AAA rupture [33].

Although we acknowledge that the aforementioned functional consequences can often be subjective, intangible, and therefore more difficult to capture reliably and at the scale of traditional clinical outcomes, patient demand suggests that these risks should also be prioritized and incorporated into the consent tools. Our tool, for example, addressed the likelihood of the patient returning home compared with the patient not returning home (ie, to a skilled care, acute care, or rehabilitation facility), incorporating an example of a procedure’s consequences to the patient’s lifestyle. Given patient feedback, and to further mirror the scope of informed consent, future iterations of surgical risk calculators should attempt to explicitly incorporate the probability of additional adverse quality-of-life outcomes and the risks associated with not pursuing a surgical intervention.

### Benefits From Using the Visual Consent Tool

The study participants did find benefits from the proposed visual consent tool, which aligns with the understanding that, generally, patient-facing decision aids have numerous benefits for patients [34]. Similar to other studies that assessed patients’ desire for risk information, the patients in our study believed that the visual consent tool has the potential to improve information retention and risk awareness [22]. However, they were concerned about how being introduced to a high number of risks might become overwhelming at times, overburden the patient, and maybe dissuade them from going through a surgery that could actually be their best option. Nevertheless, patients using similar surgical risk calculators have reported that preoperative education regarding postoperative risks actually decreases anxiety, with meta-analyses indicating that their use is associated with reduced decisional conflict and increased knowledge [34]. Although some data have shown that patients using decision aids are more likely to choose more conservative or less invasive treatments, other data have shown that the use of surgical risk calculators did not dissuade or discourage patients from pursuing surgical treatment [22,34]. These findings, combined with those of our study, support the idea that delivering risks to patients should be tailored to their needs and preferences. However, determining how many and which risks to show and when requires further research.

Along these lines, and based on the participants’ perceptions in our study, we found evidence that our visual consent tool can improve shared decision-making and be beneficial for patients and providers if appropriately customized to the particular context pertinent to the patient. This hypothesis is, of course, subject to further quantitative studies on an updated version of the visual consent tool based on the findings of this study.

### Putting the Visual Consent Tool in Broader Real-life Context

Finally, an important consideration are the stakeholders involved in incorporating the visual consent tool into the current clinical workflow. These stakeholders include the patient and their family, surgeons, and hospital administration. This study focuses on the preferences of the patients, but future work should consider input from surgeons and hospital administrators to find a solution that maximizes benefits for all. For example, although the visual consent tool exhibits benefits for the patients, some of them expressed concern that the interactions stimulated by the introduction of the visual consent tool might take too much of the surgeon’s time and negatively affect their clinical productivity. Future work should consider how to enable visual consent tool–based communication efficiency that will benefit both patients and surgeons and not significantly favor one over the other. Although the visual consent tool may disrupt current practices, it is also important to consider the greater value of such a tool to the hospital and its administration. Most patients in our study agreed that the visual consent tool can likely raise awareness, stimulate new questions, and allow them to reflect on the discussion with their surgeon after the conversation. As a consequence, the participants believed that these benefits would allow them to take a more active role in their treatment plan. For these reasons, we anticipate that the proposed visual consent tool will help promote shared decision-making by empowering patients with confidence in their decisions and attenuating the opportunities for miscommunication. Therefore, we can expect that more comprehensively informed patients will be less likely to pursue legal action when they experience a nonbeneficial outcome [5]. We believe that the proposed principles in our visual consent tool and the benefits they could bring show promise not only for patients but also for the health care system as a whole.

### Limitations

Our findings should be considered in light of the limitations of this study. The study population was biased toward older patients and only included patients who underwent a specific group of general surgeries. Their relatively positive experiences and historical exposure may have influenced their recall and opinions. Patients who undergo different surgeries, have a different demographic makeup, have worse outcomes, or are in the preoperative period may have different risk perceptions and risk visualization preferences than our study population.

Although we covered only a specific group of general surgeries, we still included a variety of them. This approach may make the results look less focused; however, it was optimal to have a setup that enabled us to learn more comprehensively about the factors that influence risk perception.

Finally, although we understand that the visual consent tool is supposed to be used with preoperative patients, for the purposes of our study, it was actually beneficial to have postoperative patients. The reason for this is that they had a chance to go through the standard consent process and the surgery and assess how different that process should have been. Once presented with the visual consent tool, they were able to evaluate how the visual consent tool might fill in the gaps in the standard consent process based on their experiences.

### Conclusions

We found that current risk calculators do not account for a number of concerns patients have, primarily related to their quality of life after the surgery, and suggest that efforts should be made to incorporate these risks into the risk calculators and the consent process. Most importantly, we identified that there is no universal way of visually communicating risks to patients, which counters the current practice of using a single approach. We found that multiple factors affect the perception of risks and that the proposed visual consent tool has the potential to provide useful information to patients and stimulate shared decision-making with their surgeons. We anticipate that these benefits can be achieved if patient characteristics are taken into account to deliver a tailored risk visualization solution. Finally, we postulate that the need for tailored visual communication of complex medical information applies to other domains of health care as well.

## Appendix

Multimedia Appendix 1 Semistructured interview guide.

## Abbreviations

AAA: abdominal aortic aneurysm
ACS: American College of Surgeons
COREQ: consolidated criteria for reporting qualitative research

## References

[1] Surgery 2015-2017 final report. National Quality Forum.

[2] Cooper Z, Courtwright A, Karlage A, Gawande A, Block S (2014). Pitfalls in communication that lead to nonbeneficial emergency surgery in elderly patients with serious illness: description of the problem and elements of a solution. Ann Surg.

[3] Angelos P (2017). The evolution of informed consent for surgery using the best case/worst case framework. JAMA Surg.

[4] Bickmore T, Utami D, Zhou S, Sidner C, Quintiliani L, Paasche-Orlow M (2015). Automated explanation of research informed consent by virtual agents. Intelligent Virtual Agents.

[5] Grauberger J, Kerezoudis P, Choudhry AJ, Alvi MA, Nassr A, Currier B, Bydon M (2017). Allegations of failure to obtain informed consent in spinal surgery medical malpractice claims. JAMA Surg.

[6] Scheer A, O'Connor AM, Chan B, Moloo H, Poulin E, Mamazza J, Auer RC, Boushey RP (2012). The myth of informed consent in rectal cancer surgery: what do patients retain?. Dis Colon Rectum.

[7] Fink AS, Prochazka AV, Henderson WG, Bartenfeld D, Nyirenda C, Webb A, Berger DH, Itani K, Whitehill T, Edwards J, Wilson M, Karsonovich C, Parmelee P (2010). Predictors of comprehension during surgical informed consent. J Am Coll Surg.

[8] Kruser JM, Nabozny MJ, Steffens NM, Brasel KJ, Campbell TC, Gaines ME, Schwarze ML (2015). "Best Case/Worst Case": qualitative evaluation of a novel communication tool for difficult in-the-moment surgical decisions. J Am Geriatr Soc.

[9] Taylor LJ, Nabozny MJ, Steffens NM, Tucholka JL, Brasel KJ, Johnson SK, Zelenski A, Rathouz PJ, Zhao Q, Kwekkeboom KL, Campbell TC, Schwarze ML (2017). A framework to improve surgeon communication in high-stakes surgical decisions: best case/worst case. JAMA Surg.

[10] Ingraham A, Agarwal S, Jung H, Liepert A, O'Rourke AP, Scarborough J (2018). Patient-centered outcome spectrum: an evidence-based framework to aid in shared decision-making. Ann Surg.

[11] Bertsimas D, Dunn J, Velmahos G, Kaafarani H (2018). Surgical risk is not linear: derivation and validation of a novel, user-friendly, and machine-learning-based predictive optimal trees in emergency surgery risk (POTTER) calculator. Ann Surg.

[12] Meguid R, Bronsert M, Juarez-Colunga E, Hammermeister K, Henderson W (2016). Surgical Risk Preoperative Assessment System (SURPAS): III. Accurate preoperative prediction of 8 adverse outcomes using 8 predictor variables. Ann Surg.

[13] Bilimoria KY, Liu Y, Paruch JL, Zhou L, Kmiecik TE, Ko CY, Cohen ME (2013). Development and evaluation of the universal ACS NSQIP surgical risk calculator: a decision aid and informed consent tool for patients and surgeons. J Am Coll Surg.

[14] Brennan M, Puri S, Ozrazgat-Baslanti T, Feng Z, Ruppert M, Hashemighouchani H, Momcilovic P, Li X, Wang DZ, Bihorac A (2019). Comparing clinical judgment with the MySurgeryRisk algorithm for preoperative risk assessment: a pilot usability study. Surgery.

[15] Tong A, Sainsbury P, Craig J (2007). Consolidated criteria for reporting qualitative research (COREQ): a 32-item checklist for interviews and focus groups. Int J Qual Health Care.

[16] Visualizing health homepage. Visualizing Health.

[17] Paling J (2003). Strategies to help patients understand risks. BMJ.

[18] Leeds IL, Rosenblum AJ, Wise PE, Watkins AC, Goldblatt MI, Haut ER, Efron JE, Johnston FM (2018). Eye of the beholder: risk calculators and barriers to adoption in surgical trainees. Surgery.

[19] Prasad KG, Nelson BG, Deig CR, Schneider AL, Moore MG (2016). ACS NSQIP risk calculator: an accurate predictor of complications in major head and neck surgery?. Otolaryngol Head Neck Surg.

[20] Vosler PS, Orsini M, Enepekides DJ, Higgins KM (2018). Predicting complications of major head and neck oncological surgery: an evaluation of the ACS NSQIP surgical risk calculator. J Otolaryngol Head Neck Surg.

[21] Lewis CM, Aloia TA, Shi W, Martin I, Lai SY, Selber JC, Hessel AC, Hanasono MM, Hutcheson KA, Robb GL, Weber RS (2016). Development and feasibility of a specialty-specific National Surgical Quality Improvement Program (NSQIP): the head and neck-reconstructive surgery NSQIP. JAMA Otolaryngol Head Neck Surg.

[22] Raymond BL, Wanderer JP, Hawkins AT, Geiger TM, Ehrenfeld JM, Stokes JW, McEvoy MD (2019). Use of the american college of surgeons national surgical quality improvement program surgical risk calculator during preoperative risk discussion. Anesthesia Analgesia.

[23] Johnson C, Campwala I, Gupta S (2017). Examining the validity of the ACS-NSQIP Risk Calculator in plastic surgery: lack of input specificity, outcome variability and imprecise risk calculations. J Investig Med.

[24] Cologne KG, Keller DS, Liwanag L, Devaraj B, Senagore AJ (2015). Use of the American College of Surgeons NSQIP surgical risk calculator for laparoscopic colectomy: how good is it and how can we improve it?. J Am Coll Surg.

[25] Etnel JR, de Groot JM, El Jabri M, Mesch A, Nobel NA, Bogers AJ, Takkenberg JJ (2020). Do risk visualizations improve the understanding of numerical risks? A randomized, investigator-blinded general population survey. Int J Med Inform.

[26] Hildon Z, Allwood D, Black N (2012). Impact of format and content of visual display of data on comprehension, choice and preference: a systematic review. Int J Qual Health Care.

[27] Kim S, Trinidad B, Mikesell L, Aakhus M (2020). Improving prognosis communication for patients facing complex medical treatment: a user-centered design approach. Int J Med Inform.

[28] van Weert JC, Alblas MC, van Dijk L, Jansen J (2021). Preference for and understanding of graphs presenting health risk information. The role of age, health literacy, numeracy and graph literacy. Patient Educ Couns.

[29] Poirier MW, Decker C, Spertus JA, McDowd JM (2019). What eye-tracking methods can reveal about the role of information format in decision-aid processing: an exploratory study. Patient Educ Couns.

[30] Pieterse AH, Finset A (2019). Shared decision making-much studied, much still unknown. Patient Educ Couns.

[31] Timmermans D, Molewijk B, Stiggelbout A, Kievit J (2004). Different formats for communicating surgical risks to patients and the effect on choice of treatment. Patient Educ Counseling.

[32] Newton-Howes PA, Bedford ND, Dobbs BR, Frizelle FA (1998). Informed consent: what do patients want to know?. N Z Med J.

[33] Williamson W, Nicoloff AD, Taylor LM, Moneta GL, Landry GJ, Porter JM (2001). Functional outcome after open repair of abdominal aortic aneurysm. J Vasc Surg.

[34] Knops A, Legemate D, Goossens A, Bossuyt P, Ubbink D (2013). Decision aids for patients facing a surgical treatment decision: a systematic review and meta-analysis. Ann Surg.

