# Supplementary material for: Effective Communication of Personalized Risks and Patient Preferences During Surgical Informed Consent Using Data Visualization: Qualitative Semistructured Interview Study With Patients After Surgery
Source: JMIR Hum Factors. 2022 Apr 29;9(2):e29118. doi: 10.2196/29118 (PMC9107059; doi:10.2196/29118)
Supplement: Multimedia Appendix 1 [file humanfactors_v9i2e29118_app1.docx]

# Semi-structured Interview Guide

*Note: The structure of this published version of the interview guide was slightly modified for better readability. No changes to the content were made.*

## I. Basic information

Age ________

Sex ________

Highest Level of Education _____________

Primary Language _____________

Surgical Procedure _____________

## II. Main Topics

1. Informed Consent

How prepared did the patient feel about what to expect from surgery?

1. Risk Visualization

How does the patient feel about our proposed solution?

1. Use of The Risk Visualization

How does the patient feel about the use of our solution in real life settings?

### Example Introduction

Hello [patient name], my name is [interviewer name]. I am a [student researcher/surgical resident] here at [Beth Israel]. Thank you so much for your interest in this research project. I will be interviewing you for about 30 minutes to learn what kinds of experience you had before and after your surgery and how we can help improve that experience. Will this be O.K. with you?

Before we begin, I have a consent form for you. [Explain briefly each section]. You are welcome to read more and ask me any questions about the form and once you’re ready, if you could sign both sheets we can begin. You can keep the documents for your reference - if any questions come up later, you will know who to contact.

This interview is split up into three sections. The first will involve some questions about your personal experience. The second will be to get your opinion on risk perception and the third will be our closing segment, which involves some final comments. I want to emphasize that this is not a test. We are here to learn from you, there are no right or wrong answers. Any answer you give will help us improve our potential solution.Informed Consent/Surgical Experience

Describe when you and your surgeon discussed your upcoming surgery.

[Potential Follow-up]

**Communicating Risks**

Do you remember any of your risks? Were there any supporting materials to show you and explain to you the risks ?

**Interpreting Risks**

Did you feel you understood your risks etc.? If not, what did you feel you didn’t understand?

What were you most concerned about for your surgery? What information was most helpful in making your decision to pursue the surgery? Why?

Is there anything you know now that you wish your surgeon would have told you beforehand?

**Participating in treatment plan**

How much did you feel you had a say in your treatment plan? Was there anything you wish your surgeon understood about your concerns, priorities and/or previous experiences?

### Risk Visualization Preferences

**Rare events**

You have two friends. One tells you that you have less than a 1% chance of getting struck by lightning. The other tells you that you have a 0.1% chance of getting struck by lightning. Do you feel differently about these two statements?

From the previous two examples, do you perceive a difference in how big of a threat it is to be struck by lightning? Which phrasing/friend do you prefer?

*If patient does not perceive a difference. Move on to show Figure 1B.*

*Otherwise show patient Figure 1A.*

Which of the following representations most clearly communicates the chance of being struck by lightning? Are the visuals helping you understand your risk of getting struck by lightning?

[Potential Follow-up] (if they cared about the numbers) Do you perceive a difference in the threat of being struck by lightning when looking at just the numbers vs. the visual? Does the visual add value compared to just seeing the numbers?

*Show patient Figure 1B*

**Common events**

You have now been told that there is a 12% chance of an earthquake today.

Which of the following representations conveys this information most clearly to you? Are the visuals helping you understand the risk of an earthquake today?

It says that you have an above average risk of an earthquake today. If that were to say below average would you feel differently about your risk of an earthquake?

## Feedback - Use of The Risk Visualization Tool

**Assess intuitiveness**

[Perception]

What kind of information do you think you can get from this visualization (picture)?

*If the patient selects A in Figure 1B present Figure 2A*

*If B present Figure 2B*

*If C present Figure 2C*

[Actionable Interpretation]

What kinds of decisions would you expect this visualization would help you make when you’re consenting for surgical procedure? [only asked if patient hasn’t already referenced whether it would impact their decision]

*Explain the purpose of the tool and the setting in which it would be used. Risk calculators exist that are able to take your patient profile e.g. age, sex, diabetes status, smoking status etc. and calculate your personalized risks for twenty complications for any surgical procedure. What this is showing is the personalized risks for a hypothetical patient. Before reaching this visualization, this hypothetical patient stated that ostomy, heart attack and stroke were of particular concern. So what this is showing are this hypothetical patients most likely complications (given their patient profile) and the hypothetical patient’s concerns as well as the chances of going home, to rehab or dying.*

**Assess the gap between the goals of the tool and the goals in reality**

*Now, understanding what we had in mind and the context of this tool.*

What aspects of this tool do you find useful? Please explain the context in which they can be useful.

**Possible use cases after full exposure to the tool**

In what setting could you imagine using this tool? OR. In what setting would you like to receive this information?

[Follow-up]

When would you want to receive the information from the tool?

Who would you want to be around at the moment when you receive the information from the tool?

**Concrete use case after full exposure to the tool**

How useful would you have found this tool when the surgeon explained your procedure to you?

Would you like to see this tool next time you have the surgeon consent you?

What aspect of the tool do you find most useful?

Do you feel this tool would allow you to engage in a deeper conversation with your surgeon regarding the risks of your surgery?

**Possible improvements of the tool**

Would there be some other information you would like to see in this final visualization?

**Closing**

Any final comments/Is there anything that you thought of during this interview that you would like to share with me?

##

## III. Figures


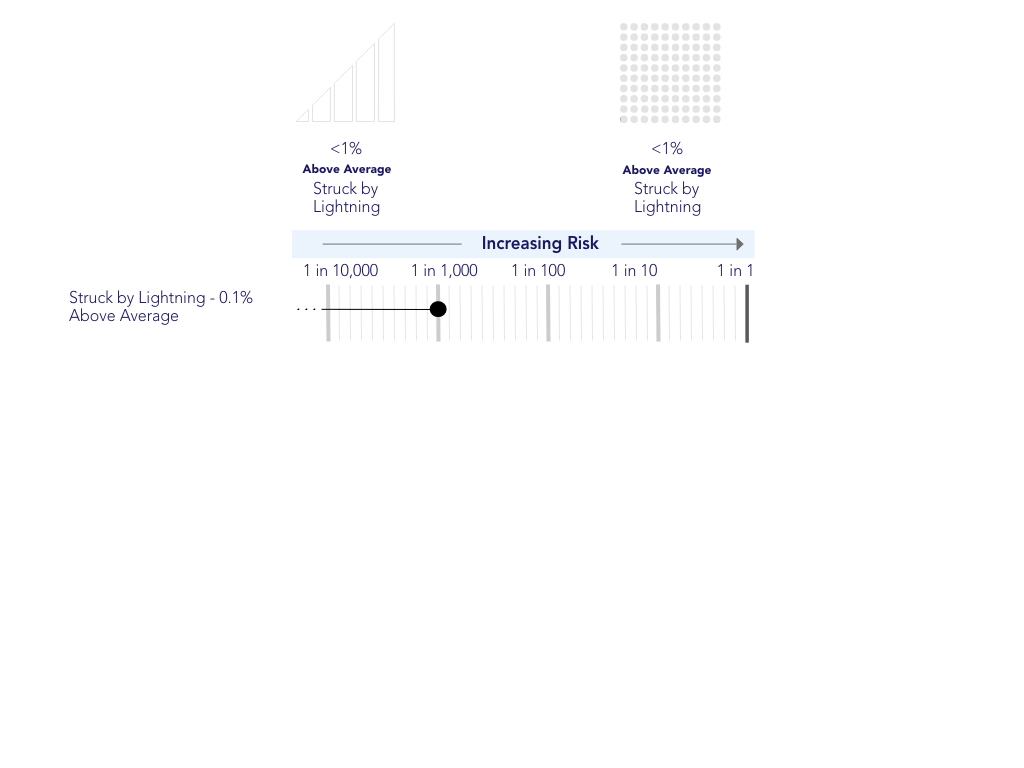


Figure 1A: Comparison of three methods for less than <1%


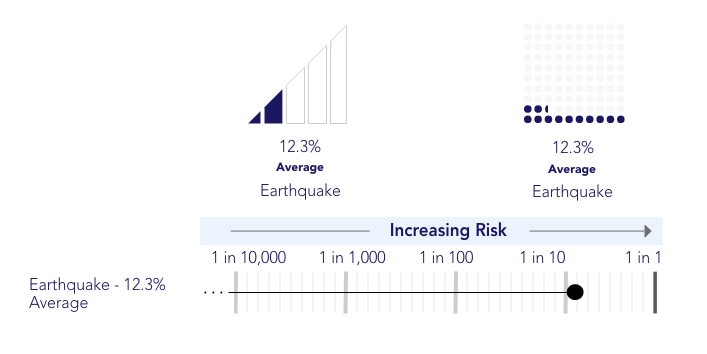


Figure 1B: Comparison of three methods at 12.3%


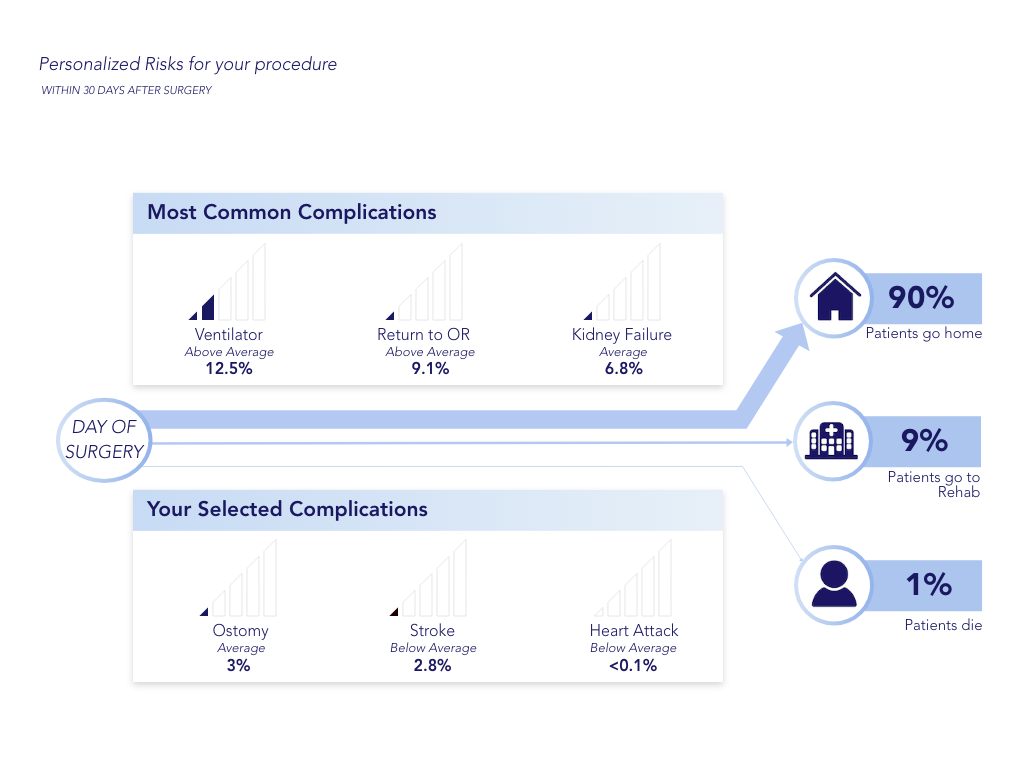


Figure 2A: Bar strength visualization of risks and discharge destination


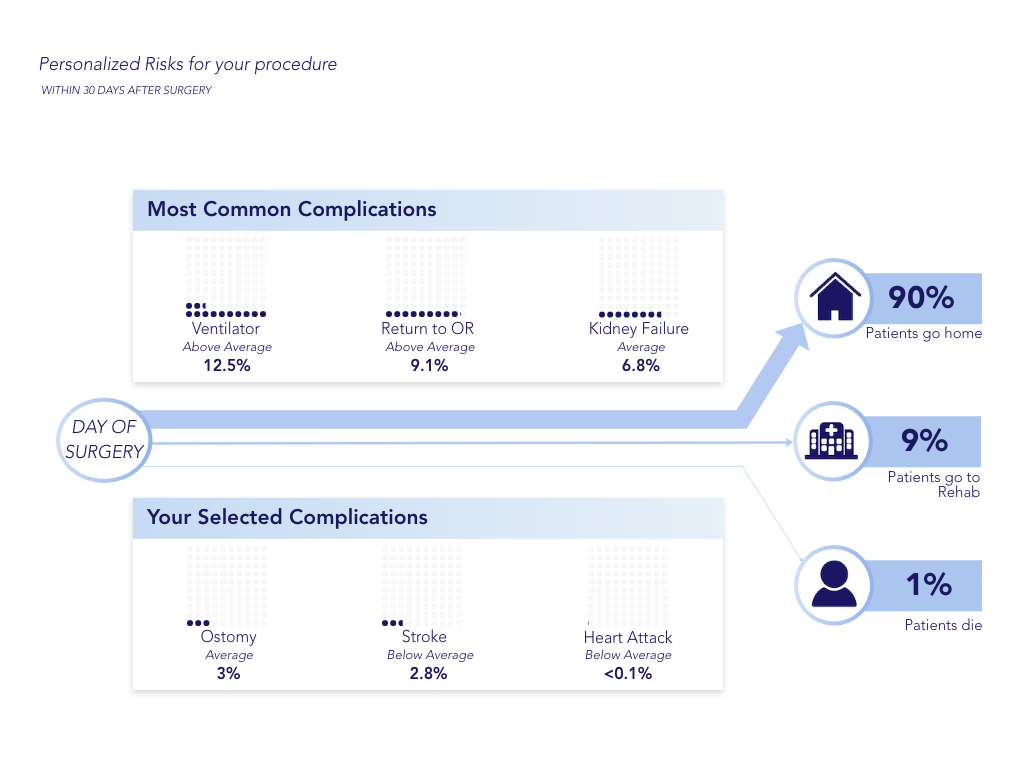


Figure 2B: Dot Array risk visualization with discharge destination


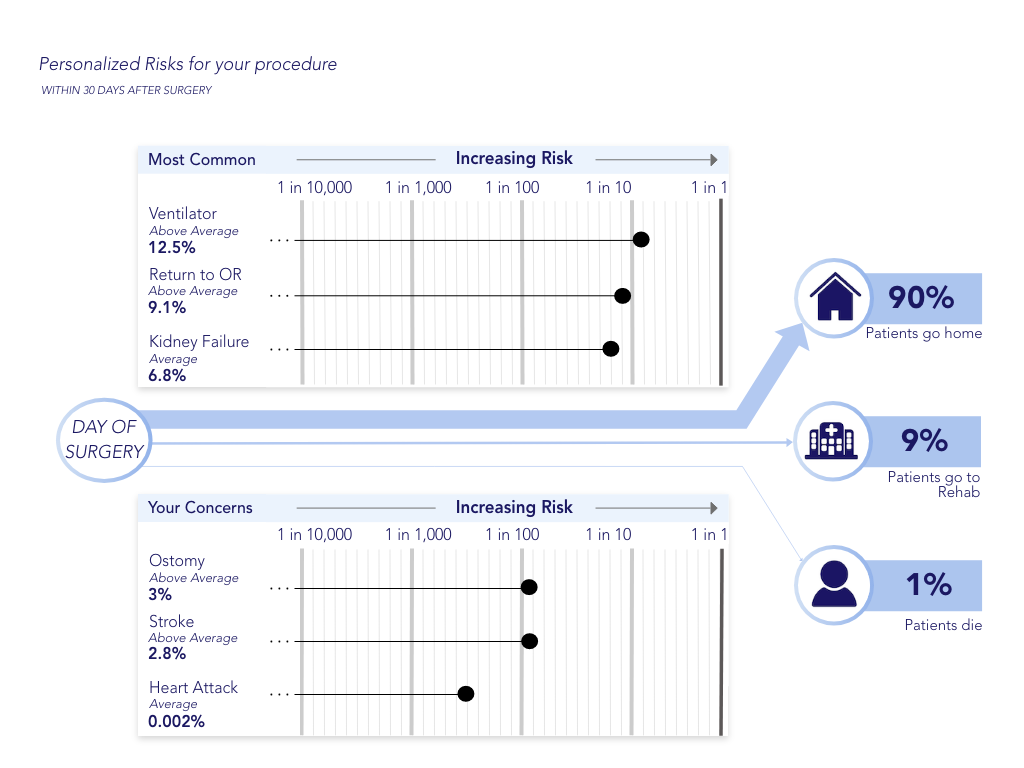


Figure 2C: Bar graph in standard format


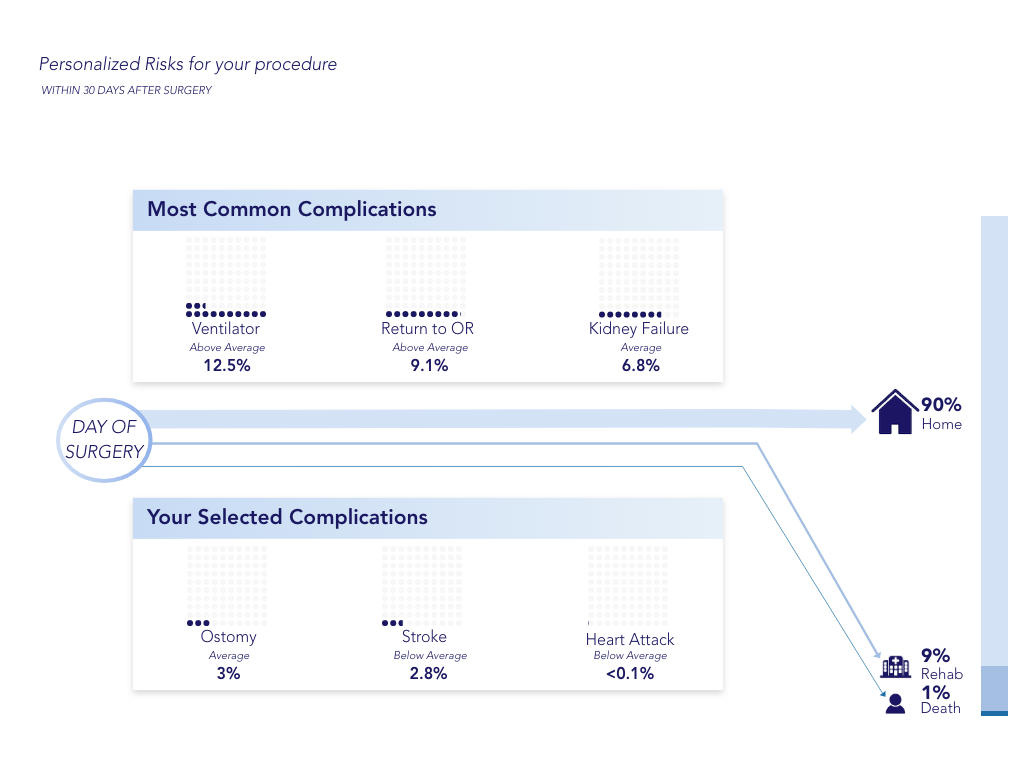


Figure 3: Discharge Destination Visualization
